# A proposed prognostic prediction score for pleuroparenchymal fibroelastosis

**DOI:** 10.1186/s12931-021-01810-z

**Published:** 2021-07-30

**Authors:** Yoshiaki Kinoshita, Takato Ikeda, Takuto Miyamura, Yusuke Ueda, Yuji Yoshida, Hisako Kushima, Masaki Fujita, Takashi Ogura, Kentaro Watanabe, Hiroshi Ishii

**Affiliations:** 1 Department of Respiratory Medicine, Fukuoka University Chikushi Hospital, 1-1-1 Zokumyoin, Chikushino, Fukuoka 818-8502 Japan; 2 Department of Respiratory Medicine, Fukuoka University Hospital, Fukuoka, Japan; 3 Department of Respiratory Medicine, Kanagawa Cardiovascular and Respiratory Center, Kanagawa, Japan; 4 Department of Respiratory Medicine, Nishi Fukuoka Hospital, Fukuoka, Japan

**Keywords:** Forced vital capacity, Pneumothorax, Krebs von den Lungen-6, Interstitial lung disease, Gender-age-physiology model

## Abstract

**Background:**

Clinical course of pleuroparenchymal fibroelastosis (PPFE) shows considerable variation among patients, but there is no established prognostic prediction model for PPFE.

**Methods:**

The prediction model was developed using retrospective data from two cohorts: our single-center cohort and a nationwide multicenter cohort involving 21 institutions. Cox regression analyses were used to identify prognostic factors. The total score was defined as the weighted sum of values for the selected variables. The performance of the prediction models was evaluated by Harrell’s concordance index (C-index). We also examined the usefulness of the gender-age-physiology (GAP) model for predicting the prognosis of PPFE patients.

**Results:**

We examined 104 patients with PPFE (52 cases from each cohort). In a multivariate Cox analysis, a lower forced vital capacity (FVC [defined as FVC < 65%]; hazard ratio [HR], 2.23), a history of pneumothorax (HR, 3.27), the presence of a lower lobe interstitial lung disease (ILD) (HR, 2.31), and higher serum Krebs von den Lungen-6 (KL-6) levels (> 550 U/mL, HR, 2.56) were significantly associated with a poor prognosis. The total score was calculated as 1 × (FVC, < 65%) + 1 × (history of pneumothorax) + 1 × (presence of lower lobe ILD) + 1 × (KL-6, > 550 U/mL). PPFE patients were divided into three groups based on the prognostic score: stage I (0–1 points), stage II (2 points), and stage III (3–4 points). The survival rates were significantly different in each stage. The GAP stage was significantly associated with the prognosis of PPFE, but no difference was found between moderate (stage II) and severe (stage III) disease. Our new model for PPFE patients (PPFE Prognosis Score) showed better performance in the prediction of mortality in comparison to the GAP model (C-index of 0.713 vs. 0.649).

**Conclusions:**

Our new model for PPFE patients could be useful for predicting their prognosis.

**Supplementary Information:**

The online version contains supplementary material available at 10.1186/s12931-021-01810-z.

## Background

Pleuroparenchymal fibroelastosis (PPFE) is a rare subtype of interstitial lung disease (ILD) that consists of elastofibrosis that is predominantly located in the upper lobes [1–5]. Clinically, patients with PPFE have some unique features that are uncommon in other ILDs, such as progressive weight loss and restrictive ventilatory impairment with increased residual volume (RV) [6–8]. The vast majority of PPFE patients die of chronic respiratory failure [9], and their 5-year survival rate was reported to be 23.3–58.9% [8, 10–12]. Although the clinical course of PPFE shows considerable variation among patients, there are no established models for predicting the prognosis of PPFE. Previous studies have suggested several promising prognostic factors, including older age [13], male sex [9, 12, 14, 15], dyspnea [12], pneumothorax events [16], lower body mass index (BMI) [17], coexistent ILD [7, 8, 10, 15, 18] or usual interstitial pneumonia (UIP) pattern in the lower lobes [8, 13, 18–20], lower elector spinae muscle attenuation on computed tomography (CT) [14], lower arterial blood oxygenation [17], lower forced vital capacity (FVC) [13, 19], lower diffusing capacity of the lung for carbon monoxide (DL_CO_) [13], and higher serum levels of Krebs von den Lungen-6 (KL-6) [8, 13, 21], and higher serum latent TGF-β binding protein-4 (LTBP-4) levels [22].

The gender-age-physiology (GAP) model has been widely used as a prognostic scoring system for patients with idiopathic pulmonary fibrosis (IPF) [23]. This model is also reported to be useful for predicting mortality in patients with chronic hypersensitivity pneumonitis, connective tissue disease-associated ILD, idiopathic nonspecific interstitial pneumonia, and unclassifiable ILD [24]. Shioya et al*.* reported that a higher GAP model was associated with poorer survival in a small retrospective cohort of patients with idiopathic PPFE [25].

The aim of this study was to develop a new prognostic prediction model for PPFE and to compare its prognostic ability with the GAP model.

## Materials and methods

### Subjects

This study was conducted with approval from the institutional review boards (Approval Numbers: 16-2-23 and C20-09-002). We retrospectively enrolled PPFE patients from two cohorts.

First, in cohort 1, we retrospectively reviewed the medical records of the Department of Respiratory Medicine at Fukuoka University Chikushi Hospital from 2000 to 2020. Consecutive patients with suspected PPFE were collected. We excluded patients who had been diagnosed with ILD other than PPFE by clinical, radiological, or histological examinations (if available). The diagnosis of PPFE (definite PPFE, radiologically and physiologically probable PPFE, and radiologically probable PPFE) was made according to the criteria proposed by Watanabe et al.[26] (Table 1).

**Table 1:** Each diagnostic category of pleuroparenchymal fibroelastosis

	Definite PPFE	Radiologically probable PPFE	Radiologically and physiologically probable PPFE
Symptom		◯	◯
Histology	◯		
Radiology	◯	◯	◯
Physiology			◯

Circles indicate the required components for the diagnosis

Symptoms: Dry cough or exertional dyspnea with insidious onset

Histology: Subpleural zonal or wedge-shaped dense fibrosis consisting of collapsed alveoli and collagen-filled alveoli with septal elastosis

Radiology: Subpleural airspace consolidation with traction bronchiectasis in upper lobes, and bilateral upward shift of hilar structures and/or volume loss in upper lobes

Physiology: RV/TLC %pred. ≥ 115% and/or BMI ≤ 20 plus RV/TLC %pred. ≥ 80%

Modified from Watanabe et al. [26]

Second, in cohort 2, patients who were enrolled in a nationwide multicenter study of PPFE conducted by the Tokyo Diffuse Lung Disease Study Group in 2015 were examined [8]. Twenty-one participating institutions presented cases with PPFE diagnosed at each institution from 2002 to 2015. The summarized clinical records and imaging and histological data were independently reviewed in advance by the core members of this project: four clinicians, two radiologists, and four pathologists. The group then held an open panel discussion on the cases. Two months later, there was an additional meeting to make a final decision on cases with a diagnosis of PPFE after multidisciplinary discussions based on the previous meeting [8].

### Clinical data

Clinical data at the diagnosis of PPFE were abstracted from the patients’ medical records. We examined the clinical background, including age, sex, underlying diseases, smoking history, history of pneumothorax before the diagnosis, and BMI; the physical examination of fine crackles and finger clubbing; laboratory findings (KL-6, surfactant protein A [SP-A], and SP-D); respiratory function parameters (FVC, the ratio of RV to total lung capacity [RV/TLC], and DLco); the six-minute walk distance, and the lowest SpO_2_. We also examined the modified Medical Research Council breathlessness scale (mMRC) and GAP score. These variables were measured at the diagnosis of PPFE.

### Radiological data

When any fibrotic lesions were observed in the lower lobes on radiological imaging, we decided whether the pattern of fibrosis was classified as a UIP pattern. In this study, the UIP pattern was defined as definite and probable UIP patterns according to the current guidelines for IPF [27], while ILD was defined as any pattern of fibrosis including the UIP pattern and PPFE. The judgment was made using CT images obtained closest to the date of the diagnosis. All images were reviewed by two observers. Interobserver disagreements were resolved by consensus. Representative chest images showing a UIP pattern and ILD in the lower lobes of PPFE patients are shown in Additional file 1: Figure S1 (Additional file 1: Table S1). Patients with PPFE have an abnormally narrowed anterior–posterior thoracic dimension (flat chest). We evaluated the flat chest index which was defined as the ratio of the anteroposterior diameter of the thoracic cage divided by the transverse diameter of the thoracic cage at the level of the sixth thoracic vertebra on a chest CT scan, as described previously [28, 29]. The measurement was made using the CT image obtained closest to the date of the diagnosis.

### Statistical analysis

All continuous variables are expressed as the mean ± standard deviation and all categorical variables are expressed as the number (percentage). Fisher’s exact test was used to compare categorical variables. For continuous variables, differences in the mean values were assessed by Student’s *t*-test for unpaired data. The survival time was defined as the period from the diagnosis to death, lung transplantation, or last contact. Survival events were defined as death or lung transplantation. The survival curves were plotted by the Kaplan–Meier method, and differences between the curves were analyzed using a log-rank test. The significance of differences between mean values was assessed by an analysis of variance followed by the Holm method for multiple comparisons [30].

We binarized the continuous variables by rounding the number of the mean value. We analyzed the relationship between these variables and the prognosis. Univariate and multivariate Cox regression analyses were used to identify prognostic factors. Stepwise selection using Akaike's information criterion (AIC) was performed for variables with *p* values of < 0.10 in a univariate analysis. A scoring system was built by proportionally weighting the regression coefficients of the prognostic factors. Each prognostic factor was multiplied by the round number of each Cox coefficient value divided by the smallest coefficient value. The total score of each patient was defined as the weighted sum of values for the selected variables. When the total score could not be determined for a patient due to missing data, the patient was excluded from the development and internal validation of a scoring system. To reduce the overfitting bias of the model, internal validation using the bootstrap method was performed 1000 times. The performance of the prediction models was evaluated by Harrell’s concordance index (C‐index). *P* values of < 0.05 were considered to indicate statistical significance. All statistical analyses were performed using R (version 4.0.3: R Foundation for Statistical Computing, Vienna, Austria).

## Results

### Patient characteristics

A total of 104 patients with PPFE (52 patients from each cohort) were eligible for inclusion in this study. The patient backgrounds of the two cohorts are summarized in Additional file 1: Table S1. Age was significantly higher in cohort 1, and the percentages of patients with a history of pneumothorax and complications of ILD and UIP in the lower lobes were higher in cohort 2. The diagnostic categories of the PPFE patients were as follows: definite PPFE (n = 55), radiologically and physiologically probable PPFE (n = 29), and radiologically probable PPFE (n = 20). The patient characteristics are summarized in Table 2. A consort diagram of the enrolled patients is shown in Additional file 1: Figure S2.

**Table 2:** Patient characteristics

Factor	n = 104
Age, years	65.3 ± 12.8
Sex, male	61 (58.7%)
Underlying disease, secondary	10 (9.61%)
Smoking history, yes	43 (42.6%)
History of pneumothorax, yes	21 (20.2%)
BMI, kg/m^2^	18.1 ± 3.05
Fine crackles, yes	44 (43.6%)
Finger clubbing, yes	8 (7.7%)
mMRC, 0/1/2/3/4 (n = 94)	24/28/25/12/5
KL-6, U/ml (n = 101)	556 ± 308
SP-A, ng/ml (n = 47)	52.2 ± 23.0
SP-D, ng/ml (n = 84)	266 ± 208
%FVC, % (n = 99)	66.1 ± 20.9
%RV/TLC, % (n = 86)	126 ± 30.7
%DL_CO_, % (n = 88)	83.0 ± 30.4
6MT distance, m (n = 51)	372 ± 148
6MT lowest SpO_2_, % (n = 58)	92.4 ± 4.24
Flat chest index	0.57 ± 0.06
UIP pattern in the lower lobes, yes	33 (31.7%)
ILD in the lower lobes, yes	68 (65.4%)

*BMI* body mass index, *mMRC* modified Medical Research Council breathlessness scale, *KL-6* Krebs von den Lungen-6, *SP* surfactant protein, *FVC* forced vital capacity, *RV* residual volume, *TLC* total lung capacity, *DL*_*CO*_ diffusing capacity of the lung for carbon monoxide, *6MT* six-minute walk test, *UIP* usual interstitial pneumonia, *ILD* interstitial lung disease

The mean age of the enrolled PPFE patients was 65.3 ± 12.8 years and 58.7% of the patients were male. More than half (57.4%) of the patients were never smokers. Ten of the 104 patients (9.61%) had underlying conditions of PPFE (ulcerative colitis [n = 3], bone marrow transplantation [n = 3], rheumatoid arthritis [n = 1], microscopic polyangiitis [n = 1], chemotherapy [n = 1], and lung transplantation for underlying lung disease [n = 1]). Enrolled patients had physiological characteristics of PPFE, including low BMI (18.1 ± 3.05 kg/m^2^), low flat chest index (0.57 ± 0.06), low %FVC (66.1 ± 20.9%), and high %RV/TLC (126 ± 30.7%). The UIP pattern was observed as a complication in 31.7% of the patients, while ILD was observed in 65.4%. The median follow‐up period was 1453 ± 1297 days and 49 patients died or underwent lung transplantation during the observation period. Twenty-one of the 104 patients (20.2%) had a history of pneumothorax before the time of the diagnosis, and the cumulative incidence of pneumothorax at the end of the observation period was 41.3% (43 of 104 patients).

**Fig. 1:**
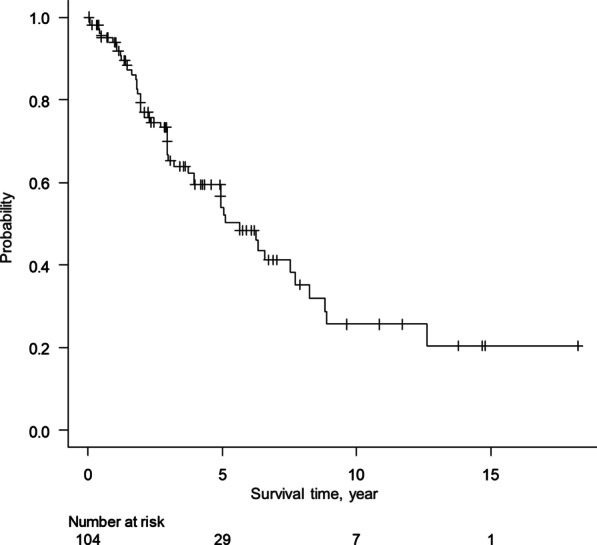
Kaplan–Meier survival curve for PPFE patients

**Fig. 2:**
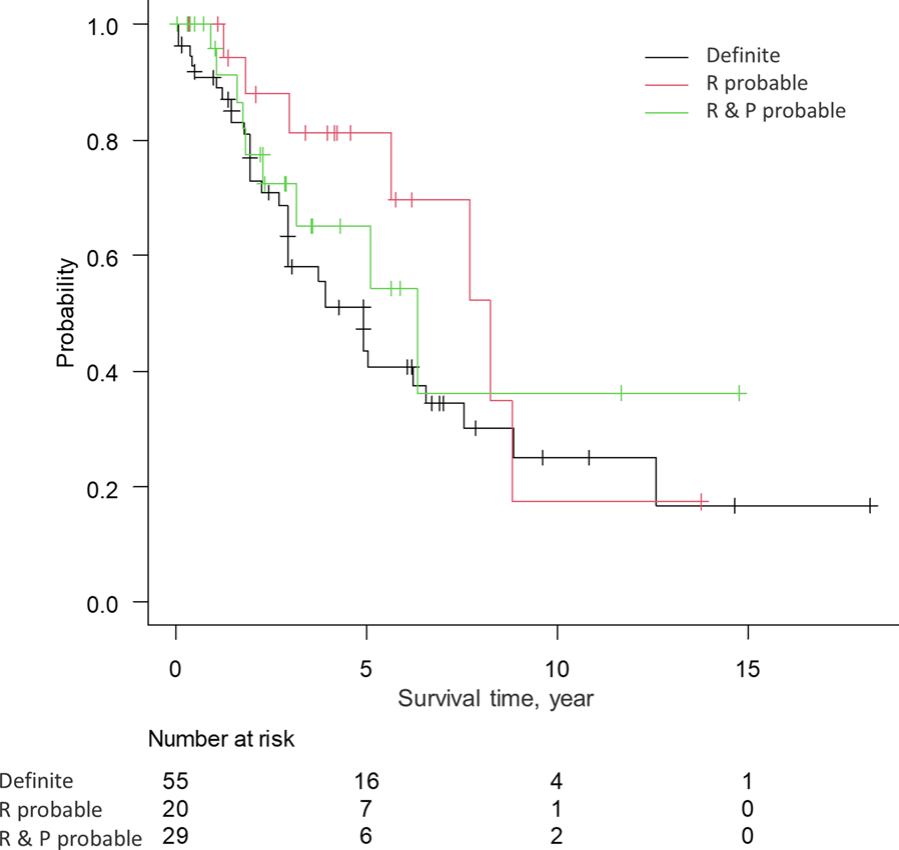
The Kaplan–Meier survival curves of PPFE patients stratified by each diagnostic category R and P probable indicates radiologically and physiologically probable PPFE, and R probable indicates radiologically probable PPFE

The 5-year overall survival rate was 54.0% (Fig. 1). The survival curves did not differ to a statistically significant extent among the diagnostic categories (definite PPFE, radiologically and physiologically probable PPFE, or radiologically probable PPFE) (Fig. 2).

### GAP stage

**Fig. 3:**
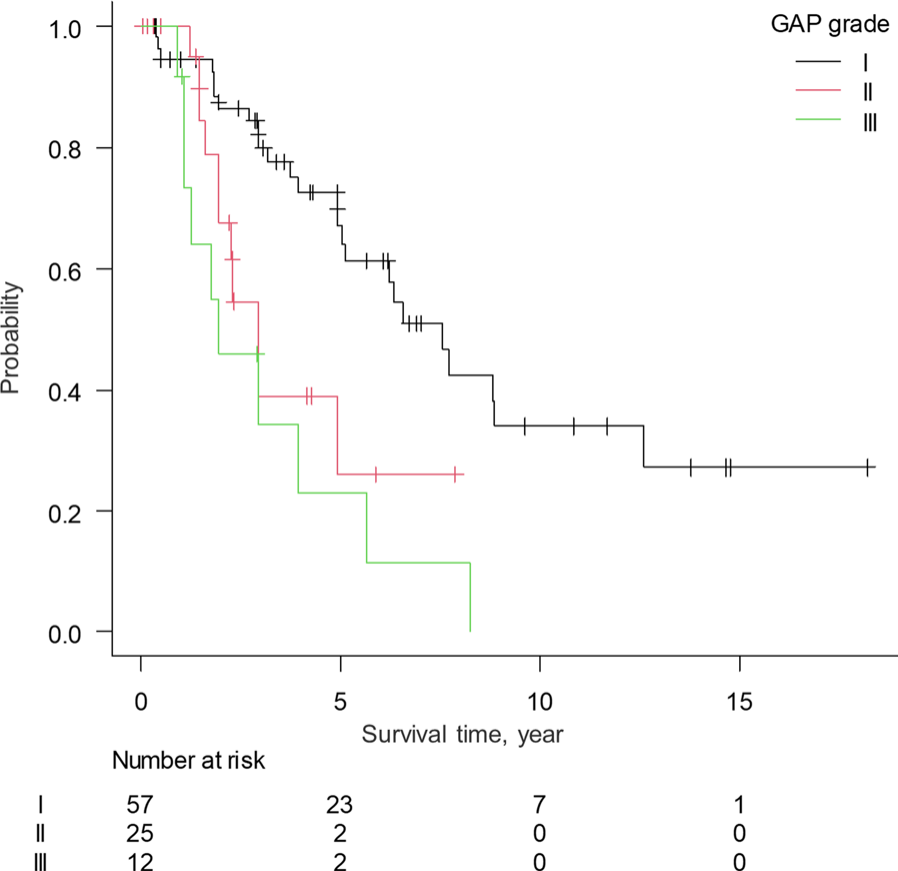
The Kaplan–Meier survival curves for PPFE patients stratified by the GAP stage

The survival curves in each GAP stage are shown in Fig. 3. The GAP stage was significantly associated with the prognosis (*p* = 0.001). Although the survival curves for the stage I patients indicated better survival in comparison to stage II (*p* = 0.025) and III (*p* < 0.001) patients, no difference was found between stage II and III patients (*p* = 0.27). The C-index of the GAP stage was 0.649.

### Cox regression analyses and the development of the prognostic prediction model

The variables were binarized as follows: age (< 65 vs. ≥ 65 years), gender (male or female), underlying disease (idiopathic or secondary), BMI (< 18 vs. ≥ 18 kg/m^2^), smoking history (yes or no), history of pneumothorax (yes or no), fine crackles (yes or no), finger clubbing (yes or no), KL-6 (< 550 vs. ≥ 550 U/ml), SP-A (< 50 vs. ≥ 50  ng/ml), SP-D (< 270 vs. ≥ 270 ng/ml), %FVC (< 65 vs. ≥ 65%), %RV/TLC (< 125 vs. ≥ 125%), %DLco (< 85 vs. ≥ 85%); six-minute walk distance (< 370 vs. ≥ 370 m), lowest SpO_2_ in six-minute walk (< 92 vs. ≥ 92%), flat chest index (< 0.57 vs. ≥ 0.57), UIP pattern in the lower lobes (yes or no), ILD in the lower lobes (yes or no), and mMRC (0–1 vs. 2–4).

The following variables were identified as significant by univariate Cox regression analyses: history of pneumothorax, low BMI, high KL-6, low %FVC, and presence of ILD in the lower lobes (Table 3). The following variables were not statistically significant but had *p* values of < 0.1: mMRC, %DL_CO_, and flat chest index (Table 3). These variables were therefore included in the stepwise selection. The following variables were finally selected: %FVC, history of pneumothorax, ILD in the lower lobes, and KL-6.

**Table 3:** Univariate Cox regression analysis

Variables	HR (95% CI)	*P* value
Age (> 65 year)	1.53 (0.85–2.76)	0.15
Gender (male)	0.88 (0.49–1.57)	0.66
Underlying disease (yes)	0.59 (0.18–1.92)	0.38
Smoking history (yes)	0.96 (0.53–1.75)	0.91
History of pneumothorax (yes)	2.98 (1.61–5.51)	< 0.001
BMI (< 18 kg/m^2^)	2.32 (1.29–4.17)	< 0.01
Fine crackles (yes)	1.38 (0.78–2.42)	0.27
Finger clubbing (yes)	0.53 (0.13–2.18)	0.38
mMRC (2–4)	1.76 (0.98–3.15)	0.058
KL-6 (> 550 U/ml)	2.62 (1.46–4.7)	< 0.01
SP-A (> 50 ng/ml)	1.34 (0.62–2.89)	0.46
SP-D (> 270 ng/ml)	1.39 (0.76–2.55)	0.29
%FVC (< 65%)	2.01 (1.12–3.59)	0.019
%RV/TLC (> 125%)	1.00 (0.52–1.92)	0.99
%DL_CO_ (< 85%)	1.99 (1.00–3.94)	0.05
6MT distance (< 270 m)	1.64 (0.76–3.56)	0.21
6MT lowest SpO_2_ (< 92%)	1.16 (0.56–2.41)	0.69
Flat chest index (< 0.57)	1.73 (0.99–3.05)	0.056
UIP pattern in the lower lobes (yes)	1.57 (0.89–2.76)	0.12
ILD in the lower lobes (yes)	2.50 (1.21–5.19)	0.014

*HR* hazard ratio, *CI* confidence interval, *BMI* body mass index, *mMRC* modified Medical Research Council breathlessness scale, *KL-6* Krebs von den Lungen-6, *SP* surfactant protein, *FVC* forced vital capacity, *RV* residual volume, *TLC* total lung capacity, *DL*_*CO*_ diffusing capacity of the lung for carbon monoxide, *6MT* six-minute walk test, *UIP* usual interstitial pneumonia, *ILD* interstitial lung disease

**Fig. 4:**
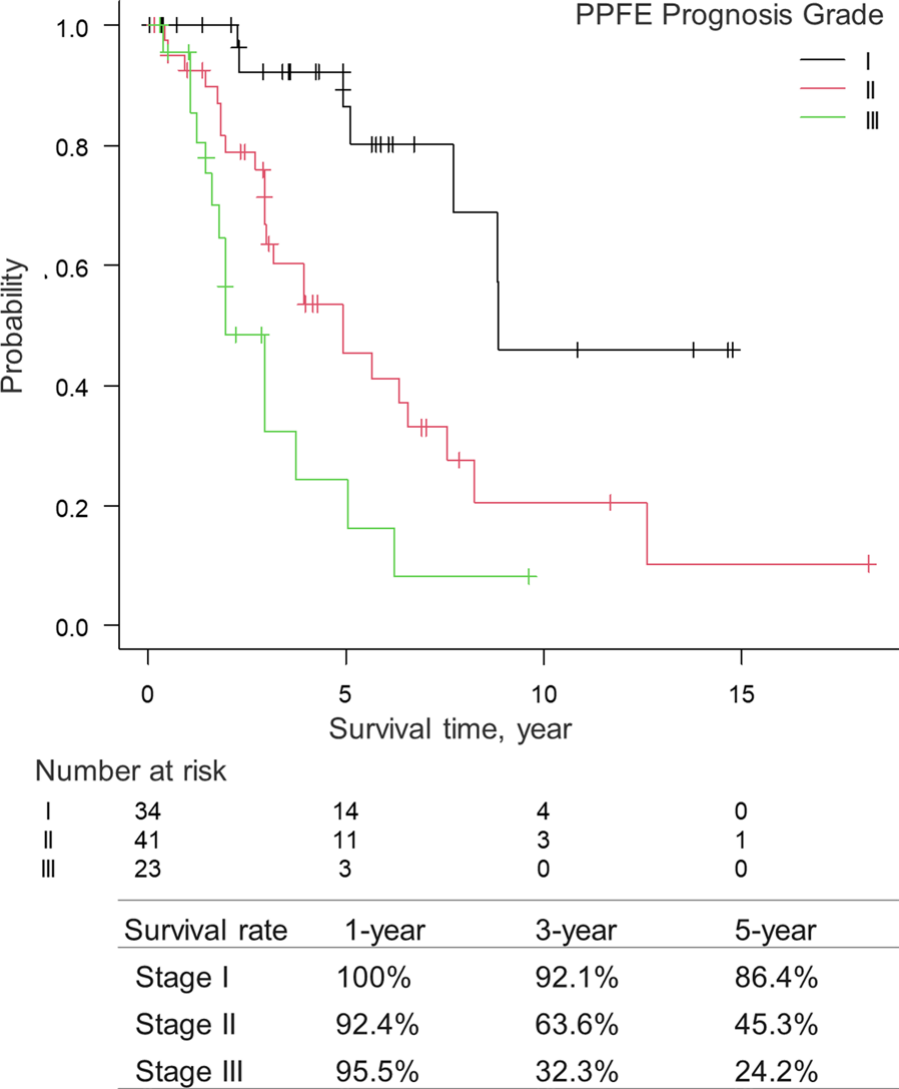
The Kaplan–Meier survival curves and survival rates in PPFE patients stratified by our prediction model

According to the coefficient values calculated by a multivariate Cox regression analysis using the selected variables (Table 4), 1 point was assigned to each factor to obtain the total score. The total score was thus calculated as 1 × (FVC, < 65%) + 1 × (history of pneumothorax, yes) + 1 × (ILD in the lower lobes, yes) + 1 × (KL-6, > 550 U/mL) (range: 0–4) (Table 5). We divided the patients into three groups based on their survival patterns: stage I (0–1 points), stage II (2 points) and stage III (3–4 points). The survival rates were significantly different in each stage (overall, *p* < 0.001; I vs. II, *p* = 0.0029; I vs. III, *p* < 0.001; II vs. III, *p* = 0.019) (Fig. 4). The new grading method (PPFE Prognosis Score) showed moderate performance in predicting the survival of PPFE patients (C-index of 0.713). The median value of the bootstrap-adjusted C-index was 0.711, which was much better than that of the GAP model (0.649). The comparison between the GAP score and the PPFE Prognosis Score is shown in Table 6.

**Table 4:** Multivariate Cox regression analysis with selected variables

Variables	HR (95% CI)	*P *value	Coefficient value	Integral coefficient
%FVC (< 65%)	2.23 (1.21–4.10)	0.01	0.801	1
History of pneumothorax (yes)	3.27 (1.68–6.38)	< 0.001	1.186	1
ILD in the lower lobes* (yes)	2.31 (1.05–5.10)	0.038	0.838	1
KL-6 (> 550 U/ml)	2.56 (1.39–4.72)	< 0.01	0.941	1

*HR* hazard ratio, *CI* confidence interval, *FVC* forced vital capacity, *ILD* interstitial lung disease, *KL-6* Krebs von den Lungen-6

*ILD in the lower lobes indicates any pattern of fibrosis including the UIP pattern and PPFE

**Table 5:** Points assigned for each variable in the prognostic prediction model

Variables	Classification	Points
%FVC (< 65%)	Yes	1
	No	0
History of pneumothorax	Yes	1
	No	0
ILD in the lower lobes*	Yes	1
	No	0
KL-6 (> 550U/ml)	Yes	1
	No	0
Stage	Grade	
	I	0–1
	II	2
	III	3–4

*FVC* forced vital capacity, *ILD* interstitial lung disease, *KL-6* Krebs von den Lungen-6

*ILD in the lower lobes indicates any pattern of fibrosis including the UIP pattern and PPFE

**Table 6:** The comparison between GAP score and PPFE prognosis score

Predictor	GAP score	PPFE prognosis score
Gender	female (0), male (1)	–
Age, year	≤ 60 (0), 61–65 (1), > 65 (2)	–
FVC, % predicted	> 75 (0), 50–75 (1), < 50 (2)	≥ 65 (0), < 65 (1)
DL_CO_, % predicted	> 55 (0), 36–55 (1), ≤ 35 (2), cannot perform (3)	–
History of pneumothorax	–	No (0), yes (1)
ILD in the lower lobes*	–	No (0), yes (1)
KL-6, U/ml	–	≤ 550 (0), > 550 (1)
Total points	I (0–3), II (4–5), III (6–8)	I (0–1), II (2), III (3–4)

The figures in parentheses indicate the corresponding points

*GAP* gender-age-physiology, *FVC* forced vital capacity, *DL*_*CO*_ diffusing capacity of the lung for carbon monoxide, *ILD* interstitial lung disease, *KL-6* Krebs von den Lungen-6

*ILD in the lower lobes indicates any pattern of fibrosis including the UIP pattern and PPFE

## Discussion

We developed a new prognostic prediction model (PPFE Prognosis Score) for PPFE patients, which uses the following variables: FVC, history of pneumothorax, ILD in the lower lobes, and serum KL-6 level. This model demonstrated better performance in predicting the mortality of PPFE patients than the GAP model.

The GAP stage was significantly associated with the prognosis of PPFE but was not sensitive for distinguishing between moderate (stage II) and severe (stage III) disease. The GAP model is a useful scoring system for predicting the prognosis in various types of ILD [23, 24]. PPFE has two different radiological and physiological aspects: pulmonary fibrosis and chest wall deformity [1–5, 31, 32]. PPFE differs from the majority of ILDs in that the elastofibrosis in PPFE is predominantly located in the upper lobes [1–5, 31]. In addition, chest wall abnormality (platythorax or flattened thoracic cage) is a unique characteristic of PPFE [28, 29, 31, 32]. Thus, the prognostic factors or prognostic prediction models for PPFE may differ from other ILDs.

Using our proposed simple prognostic model (PPFE Prognosis Score), the prognosis of PPFE patients was significantly stratified at each stage. The following factors were included in our model: FVC, history of pneumothorax, ILD in the lower lobes, and serum KL-6 levels. Kono et al*.* [19] examined 89 patients with idiopathic PPFE and showed that low %FVC and the coexistence of lower-lobe ILD on CT, especially the UIP pattern, may predict poor survival in patients with idiopathic PPFE. Recently, Oda et al*.* [13] conducted a study of 164 patients with PPFE, which has been the largest cohort to date, and showed that age, FVC, DLco, KL-6, and the complication of UIP were independent prognostic factors for patients with PPFE [13]. These studies suggested that low %FVC and the coexistence of UIP are strong predictors of survival in patients with PPFE.

We hypothesize that the prognosis of PPFE patients complicated with ILD including UIP is poorer than that in patients without ILD due to the presence of their progressive fibrosis in both the upper and lower lobes. UIP is the most common pattern of ILD in the lower lobes of PPFE patients [7, 10, 33], and some studies showed that coexistent UIP is associated with a poor outcome in PPFE patients [8, 18, 19]. In our study, fewer patients were complicated with the UIP pattern in comparison to the ILD pattern (33/104 cases vs. 68/104 cases). Coexistent UIP was not associated with a poor outcome in PPFE patients possibly because of the small sample size. Meanwhile, Enomoto et al. [10] showed that there was no significant difference in the prognosis between PPFE patients with a UIP pattern and those with other fibrosis. Therefore, the pattern of fibrosis (UIP or ILD) that should be included in the prognostic model for PPFE may change in future studies.

A prior history of pneumothorax at the time of the diagnosis was included in our prognosis prediction model for PPFE. Pneumothorax is one of the major complications in PPFE patients, and pneumothorax in patients with PPFE is sometimes recurrent and untreatable [1, 2, 6, 10, 33–36]. The importance of pneumothorax as a prognostic factor in PPFE patients has rarely been examined. Kono et al*.* [16] examined 89 patients with idiopathic PPFE and showed that a prior history of pneumothorax is significantly associated with poorer outcomes. The incidence of pneumothorax might be higher in PPFE patients with advanced stages than those with early stages. In this study, the incidence of pneumothorax at the time of the diagnosis was not very high (20.2%); however, it increased to 41.3% at the end of the observation period. Tanizawa et al. [37] reported that 80% of patients with PPFE had a history of pneumothorax at the time of registration for lung transplantation. Therefore, a history of pneumothorax could reflect the severity of the disease in PPFE patients.

Several serum biomarkers (e.g., KL-6, SP-A, and SP-D) are useful in the diagnosis of ILD [38]. Among them, serum KL-6 is the most sensitive biomarker in predicting the prognosis of IPF [39, 40]. Some studies have shown that high serum KL-6 levels are associated with a poor prognosis in patients with PPFE [8, 13, 21]. Fibrotic lesions in patients with PPFE begin in the upper lobes and progress to the lower lobes [8, 41]. Serum KL-6 is usually within the normal range or around the upper normal limit in PPFE patients without ILD in the lower lobes [8, 41]. The more frequent association of ILD in the lower lobes suggests that coexisting non-PPFE fibrosing ILD is the main cause of the elevated levels of serum KL-6 in advanced-stage PPFE [8]. Therefore, it is reasonable to consider that higher serum KL-6 levels are associated with a poorer prognosis in PPFE patients.

The present study was associated with some limitations. Although considering the rarity of PPFE, this study had a significant number of cases, the sample size was too small to perform an external validation analysis of the model. Second, treatment was not considered in our model. No treatments have been proven to be effective for PPFE. Nintedanib, a tyrosine kinase inhibitor, has become available for progressive fibrosing ILD and may affect the prognosis of the enrolled patients. However, the impact of the drug is negligible because the use of nintedanib for progressive fibrosing ILD was started in Japan in 2020. In addition, a retrospective study showed that the efficacy of antifibrotic agents was limited in PPFE patients with UIP [42]. Third, we have recently reported that high serum LTBP-4 levels may be associated with a poor prognosis in PPFE patients [22]; however, serum LTBP-4 was not measured in the present study.

## Conclusion

In conclusion, the results of the present study suggest that our new model for PPFE patients could be useful for predicting their prognosis.

## Supplementary Information

**Additional file 1. Table S1.** Patient characteristics in each cohort. **Figure S1**. UIP pattern and ILD in the lower lobes. **Figure S2**. Consort diagram of the enrolled patients.

## Data Availability

All data generated or analyzed during this study are included in this published article and its supplementary information files. The datasets used and/or analyzed during the current study are available from the corresponding author on request.

## Abbreviations

AIC: Akaike's information criterion
BMI: body mass index
CT: computed tomography
DL_CO_: diffusing capacity of the lung for carbon monoxide
FVC: forced vital capacity
GAP model: gender-age-physiology model
HR: hazard ratio
ILD: interstitial lung disease
IPF: idiopathic pulmonary fibrosis
KL-6: Krebs von den Lungen-6
LTBP-4: latent TGF-β binding protein-4
mMRC scale: modified Medical Research Council breathlessness scale
PPFE: pleuroparenchymal fibroelastosis
RV: residual volume
SP: surfactant protein
TLC: total lung capacity
UIP: usual interstitial pneumonia
