# Supplementary material for: A proposed prognostic prediction score for pleuroparenchymal fibroelastosis
Source: Respir Res. 2021 Jul 30;22:215. doi: 10.1186/s12931-021-01810-z (PMC8400711; doi:10.1186/s12931-021-01810-z)

**Additional Table S1. Patient characteristics in each cohort**

| Factor | Cohort 1 (n = 52) | Cohort 2 (n = 52) | P-value |
| --- | --- | --- | --- |
| Age, years | 70.6 ± 9.52 | 60.1 ± 13.6 | <0.001 |
| Sex, male | 30 (57.7%) | 31 (59.6%) | 1 |
| Underlying disease, secondary | 6 (11.5%) | 4 (7.7%) | 0.741 |
| Smoking history, yes | 22 (42.3%) | 21 (42.9%) | 1 |
| History of pneumothorax, yes | 5 (9.6%) | 16 (30.8%) | 0.013 |
| BMI, kg/m^2^ | 18.2 ± 3.36 | 18.0 ± 2.73 | 0.83 |
| Fine crackles, yes | 20 (40.8%) | 24 (46.2%) | 0.689 |
| Finger clubbing, yes | 6 (11.5%) | 2 (3.8%) | 0.269 |
| mMRC, 0/1/2/3/4 | 8/12/13/8/1 | 16/16/12/4/4 | 0.272 |
| KL-6, U/ml | 516 ± 315 | 594 ± 299 | 0.207 |
| SP-A, ng/ml | 57.9 ± 25.7 | 48.4 ± 20.6 | 0.167 |
| SP-D, ng/ml | 255 ± 141 | 274 ± 245 | 0.677 |
| %FVC, % | 67.0 ± 22.4 | 65.3 ± 19.5 | 0.7 |
| %RV/TLC, % | 120 ± 34.4 | 132 ± 25.7 | 0.091 |
| %DL_CO_, % | 92.8 ± 30.0 | 74.9 ± 28.5 | 0.008 |
| 6MT distance, m | 291 ± 101 | 438 ± 150 | <0.001 |
| 6MT lowest SpO_2_, % | 92.4 ± 5.32 | 92.4 ± 3.43 | 0.965 |
| Flat chest index | 0.58 ± 0.07 | 0.56 ± 0.06 | 0.367 |
| UIP pattern in the lower lobes, yes | 10 (19.2%) | 23 (44.2%) | 0.011 |
| ILD in the lower lobes, yes | 25 (48.1%) | 43 (82.7%) | <0.001 |

BMI, body mass index; mMRC, modified Medical Research Council breathlessness scale; KL-6, Krebs von den Lungen-6; SP, surfactant protein; FVC, forced vital capacity; RV, residual volume; TLC, total lung capacity; DL_CO_, diffusing capacity of the lung for carbon monoxide; 6MT, six-minute walk test; UIP, usual interstitial pneumonia; ILD, interstitial lung disease.

**Additional Figure S1.** **UIP pattern and ILD in the lower lobes**


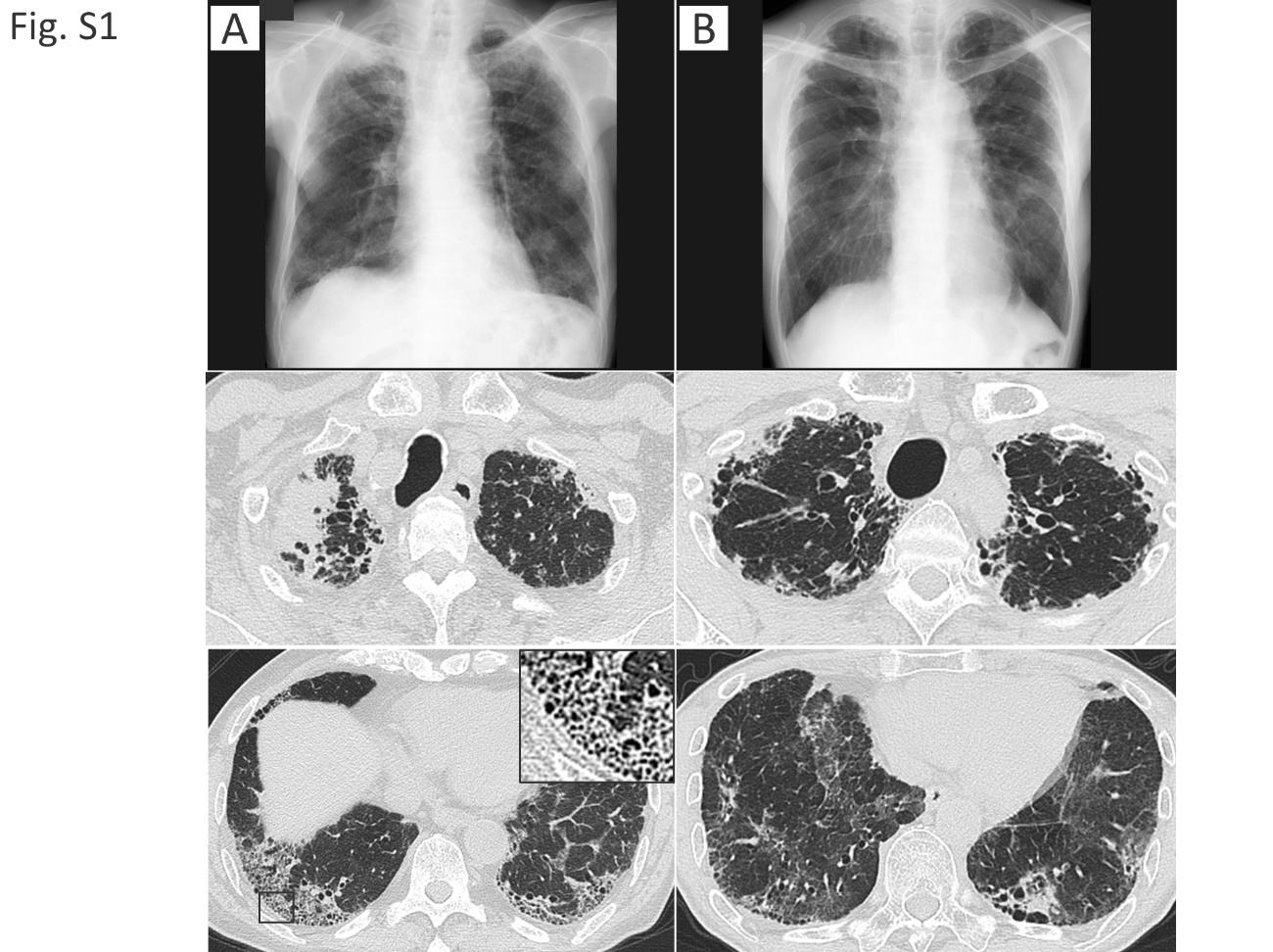


Chest radiography and computed tomography in PPFE patients complicated with interstitial lung disease (ILD) in the lower lobes (A and B). Both patients had upper-lobe-dominant consolidation in the bilateral lung apexes. Honeycomb fibrosis was evident (inset of Fig. A), and the fibrotic lesion in the lower lobes of Fig. A was classified as a usual interstitial pneumonia (UIP) pattern. The fibrotic lesion in the lower lobes of Fig. B was classified as an alternative diagnosis for UIP because of the extensive ground-glass attenuation.

**Additional Figure S2. Consort diagram of the enrolled patients**


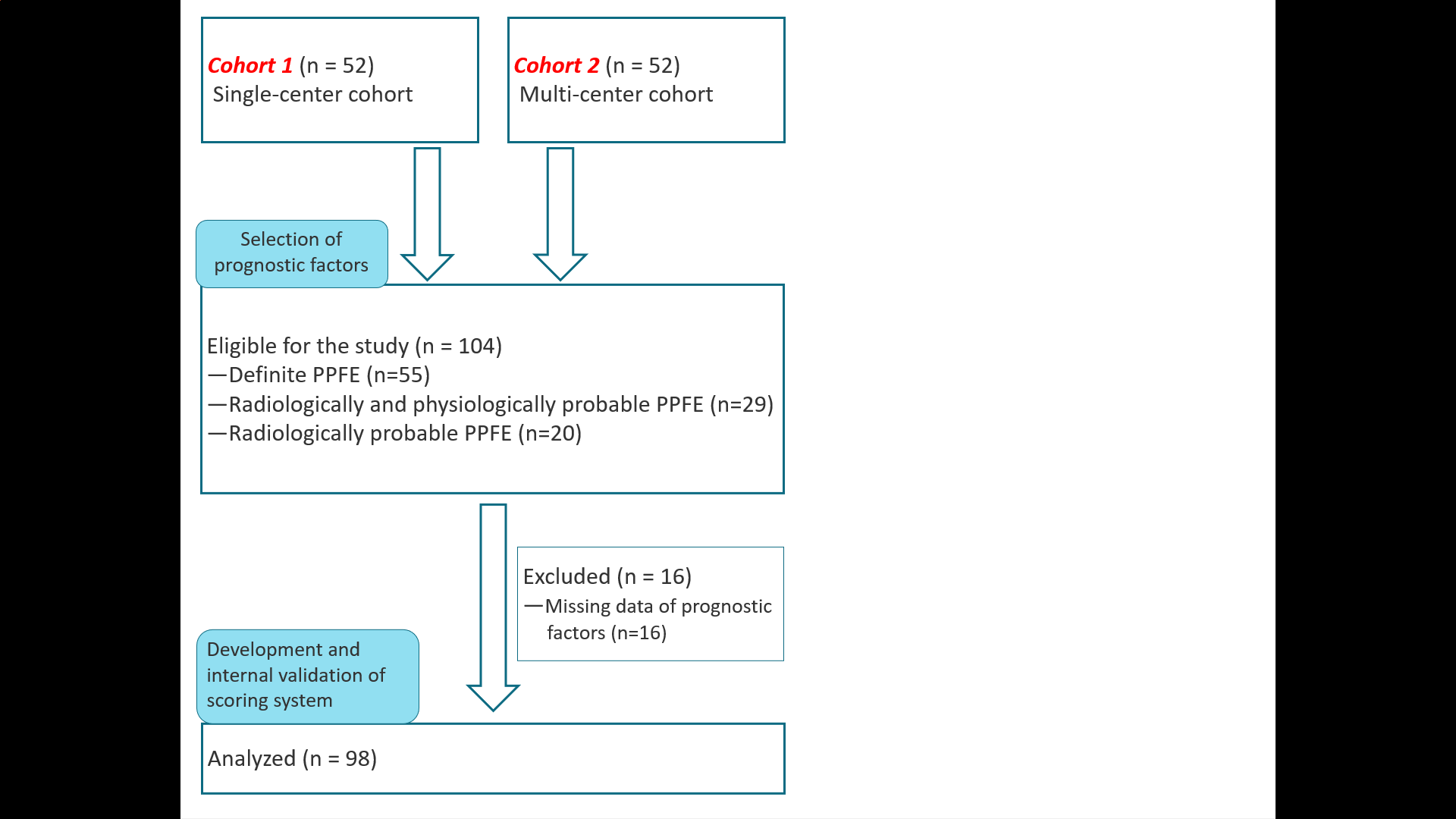

Supplement: Supplementary file 1 — Additional file 1. Table S1. Patient characteristics in each cohort. Figure S1. UIP pattern and ILD in the lower lobes. Figure S2. Consort diagram of the enrolled patients. [file 12931_2021_1810_MOESM1_ESM.docx]
